# *QuickStats:* Age-Adjusted Death Rates* for Cancer, by Urban-Rural Status^†^ and Sex — National Vital Statistics System, United States, 1999–2019

**DOI:** 10.15585/mmwr.mm7037a8

**Published:** 2021-09-17

**Figure:**
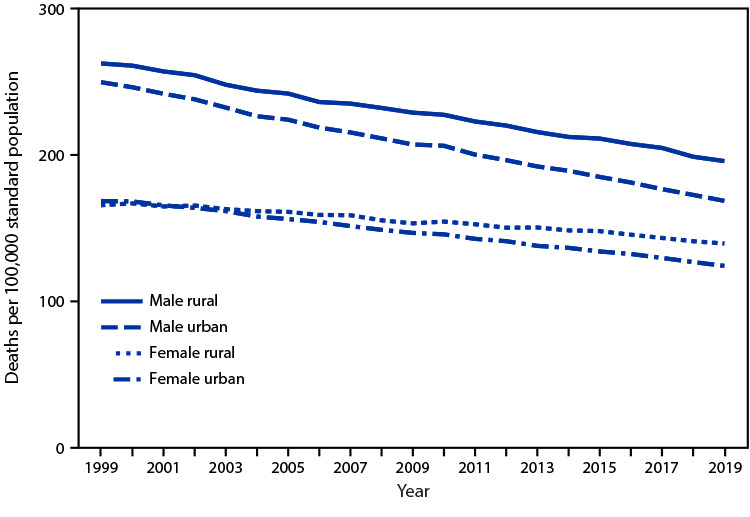
Cancer death rates declined among males and females during 1999–2019 in urban areas from 249.6 per 100,000 to 168.4 for males and from 168.2 to 123.9 for females. Rates also declined in rural areas from 262.4 to 195.6 for males and from 165.4 to 139.2 for females. Throughout the period, cancer death rates were higher for males than females and in rural compared with urban areas, and the urban-rural differences widened over the period for both males and females.

**Source:** National Center for Health Statistics, National Vital Statistics System, Mortality Data. https://www.cdc.gov/nchs/nvss/deaths.htm

For more information on this topic, CDC recommends the following link: https://www.cdc.gov/cancer/dcpc/prevention/

